# Chronic exposure of colorectal cancer cells to bevacizumab promotes compensatory pathways that mediate tumour cell migration

**DOI:** 10.1038/bjc.2011.81

**Published:** 2011-03-15

**Authors:** F Fan, S Samuel, P Gaur, J Lu, N A Dallas, L Xia, D Bose, V Ramachandran, L M Ellis

**Affiliations:** 1 Department of Cancer Biology, The University of Texas MD Anderson Cancer Center, Unit 173, PO Box 301402, Houston, TX 77030-1402, USA; 2 Department of Surgical Oncology, The University of Texas MD Anderson Cancer Center, Unit 173, PO Box 301402, Houston, TX 77030-1402, USA

**Keywords:** bevacizumab, VEGF, colorectal cancer, resistance, migration, metastasis

## Abstract

**Background:**

Bevacizumab (Bev), a monoclonal antibody to vascular endothelial growth factor (VEGF), is used in combination with chemotherapy for the treatment of metastatic colorectal cancer (CRC). The effects of Bev on angiogenesis have been well described, but the direct effect of Bev on tumour cells is unknown. This study was carried out to determine the molecular and phenotypic changes in CRC cells after chronic Bev exposure *in vitro.*

**Methods:**

Human CRC cell lines were chronically exposed (3 months) to Bev *in vitro* to develop Bev-adapted (Bev-A) cell lines. Vascular endothelial growth factor family members were determined by reverse transcription–polymerase chain reaction and western blotting. Migration and invasion was determined using standard *in vitro* assays. Intravenous injection of tumour cells was carried out to evaluate metastatic potential in mice.

**Results:**

Bevacizumab-adapted cells were found to be more migratory and invasive than control cells (*P*<0.001). Bevacizumab-adapted cells showed higher levels of VEGF-A, -B, -C, placental growth factor (PlGF), VEGF receptor-1 (VEGFR-1) and phosphorylation of VEGFR-1. Furthermore, treatment with SU5416, a VEGFR protein tyrosine kinase inhibitor, led to significantly decreased cell migration *in vitro* (*P*<0.001). Bevacizumab-adapted cells were more metastatic *in vivo* (*P*<0.05).

**Conclusion:**

Chronic exposure of CRC cells to Bev (1) increased expression of VEGF-A, -B, -C, PlGF, VEGFR-1 and VEGFR-1 phosphorylation, (2) increased tumour cell migration and invasion, and (3) metastatic potential *in vivo*. Our study shows the functional significance of autocrine VEGF signalling in CRC cells.

Angiogenesis is a fundamental event in the process of tumour growth and metastatic dissemination. The well-established role of VEGF in promoting tumour angiogenesis and the pathogenesis of human cancers has led to the development of therapeutic strategies that selectively target this pathway. The FDA-approved anti-VEGF antibody bevacizumab (Bev) is one such validated antiangiogenic therapeutic agent that, when used in combination with chemotherapy, has been shown to prolong survival in patients with metastatic colorectal cancer (CRC) (Hurwitz *et al*, 2004). However, it has become clear that virtually all patients, regardless of their tumour type, will ultimately exhibit disease progression while on VEGF-targeted therapy. Therefore, understanding the mechanisms by which tumours adapt to VEGF blockade is important in optimising therapeutic regimens utilising this approach.

The role of VEGF in the process of tumour angiogenesis, by stimulation of vascular endothelial growth factor receptors (VEGFRs) on the tumour endothelium, is well established (reviewed in Hicklin and Ellis (2005) and Ferrara (2009)). However, over the past decade, studies have shown that VEGFRs are also present on numerous cell types, including haematopoietic progenitor cells (Kaplan *et al*, 2005), cells of the central nervous system (Poesen *et al*, 2008), immune mediator cells (Shin *et al*, 2009) and tumour cells themselves (Fan *et al*, 2005; Wey *et al*, 2005). Our laboratory has shown that VEGF can stimulate tumour cell migration and invasion of CRC cells (Fan *et al*, 2005), and these processes were thought to be mediated through VEGFR-1 as a specific antibody (18F1) to VEGFR-1 blocked VEGF-mediated responses (Fan *et al*, 2005). More recent studies from our laboratory have focused on the role of the VEGF co-receptor, neuropilin-2 (NRP-2), which mediates tumour cell survival pathways (Dallas *et al*, 2008). Thus, it has become increasingly clear that tumour cells express VEGFRs, and thus it is possible that some of the effects observed with anti-VEGF therapies are due to direct effects on tumour cells.

As VEGFRs are present and functional on CRC cells, it is possible that anti-VEGF therapy could inhibit processes mediated by these receptors. On the other hand, it is also possible that, over the long term, tumour cells could adapt (become resistant) to the inhibition of VEGF signalling and therefore their phenotype could be altered. As has been shown in mice and human beings, administration of anti-VEGF therapy initiates compensatory pathway activation, including upregulation of placental growth factor (PlGF) and stromal-derived factor-1 (SDF-1) (Batchelor *et al*, 2007; Ebos *et al*, 2007; Folkins *et al*, 2009). In several recent studies, investigators showed that *in vivo* administration of VEGF-targeted therapies could lead to an increase in metastasis, but the exact mechanism, and the cell types mediating this mechanism, has yet to be identified (Ebos *et al*, 2009; Paez-Ribes *et al*, 2009).

In this study, we investigated the molecular and phenotypic changes associated with chronic exposure of CRC cells to Bev *in vitro*. We found that chronic exposure of CRC cells to Bev leads to increased migration and invasion *in vitro* that is associated with increased expression of alternate VEGF family ligands, PlGF and VEGF-C, and activation of VEGFR-1. Inhibition of activation of VEGFRs blocked the increase in migration observed in Bev-adapted cells. Bevacizumab-adapted cells exhibited an increase in metastatic potential *in vivo*. These results provide new mechanistic insights into the phenotypic alterations associated with Bev adaptation of CRC cells. Studies such as these will help dissect out the relative effects of chronic exposure of Bev on tumour cells, elucidating the effect of VEGF inhibition on the numerous individual components of the tumour microenvironment.

## Materials And Methods

### Reagents and antibodies

Bevacizumab (Genentech BioOncology, South San Francisco, CA, USA) was obtained from the pharmacy at MD Anderson Cancer Center. SU5416 was purchased from Sigma-Aldrich (St Louis, MO, USA). Antibodies for western blot analysis were as follows: polyclonal goat anti-VEGF-A, polyclonal goat anti-VEGF-B_167/186_ (R&D Systems Inc., Minneapolis, MN, USA), rabbit anti-VEGF-C (Zymed Laboratories, San Francisco, CA, USA), polyclonal rabbit anti-PlGF (Abcam, Cambridge, MA, USA), polyclonal rabbit anti-phospho-VEGFR-1 antibody (Millipore Corporation, Billerica, MA, USA), polyclonal rabbit anti-Flt-1 (VEGFR-1), polyclonal rabbit anti-Phospho-VEGFR-2 antibody and polyclonal rabbit anti-VEGFR-2 (Cell Signaling, Danvers, MA, USA), polyclonal rabbit anti-phospho-VEGFR-2 (Novus, Littleton, CO, USA), polyclonal rabbit anti-VEGFR-3, NRP-1 and mouse monoclonal NRP-2, Nonspecific mouse IgG (Santa Cruz Biotechnology Inc., Santa Cruz, CA, USA).

### Cell lines and culture conditions

HCT116 and SW480 human CRC cell lines were obtained from the American Type Culture Collection (Manassas, VA, USA). Cell lines were cultured in minimal essential medium (MEM) supplemented with 5% foetal bovine serum (FBS), penicillin–streptomycin, vitamins, sodium pyruvate, L-glutamine and non-essential amino acids (Life Technologies, Grand Island, NY, USA) at 37°C in 5% CO_2_ and 95% air. Cells were confirmed to be free of mycoplasma using the ‘MycoAlert’ Mycoplasma Detection Kit (Lonza Group, Basel, Switzerland). Results from all *in vitro* studies were confirmed in at least three independent experiments to verify results. All the experiments were performed when cells reached 50–60% confluence.

### Development of Bev-adapted CRC cells

The human CRC cell lines HCT116 and SW480 were exposed to a clinically relevant dose of Bev (250 *μ*g ml^−1^) (Herbst *et al*, 2005) for 3 months *in vitro* to develop the Bev-adapted (Bev-A) cell lines HCT116/Bev-A and SW480/Bev-A. HCT116 and SW480 cells were also exposed to mouse IgG (250 *μ*g ml^−1^) in parallel to generate the control cell lines HCT116/control and SW480/control.

### Reverse transcription–polymerase chain reaction

Polymerase chain reaction (PCR) amplification of VEGFR-1,-2, -3, NRP-1 and -2 was performed under the following conditions: 95°C for 5 min, 27–40 cycles of 45 s denaturing at 95°C, 45 s of annealing at 57°C and 1 min of extension at 72°C. Products were analysed by electrophoresis of 20 *μ*l of each PCR reaction mixture in a 1.5% agarose gel, and bands were visualised by ethidium bromide staining. Human umbilical vein endothelial cells served as a positive control. The following primers were used: VEGFR-1 (550 bp, 40 cycles) – sense primer, 5′-GACCTGGAGTTACCCTGA-3′ and antisense primer, 5′-GACACGGCCTTTTCGTA-3′ VEGFR-2 primer (777 bp, 40 cycles) – sense primer, 5′-CTGGCGGCACGAAATATCCTCTTA-3′ and antisense primer, 5′-GGGCACCATTCCACCAAAAGAT-3′ VEGFR-3 (381 bp, 40 cycles) – sense primer, 5′-CCCACGCAGACATCAAGACG-3′ and antisense primer, 5′-TGCAGAACTCCACGATCACC-3′ NRP-1 (450 bp, 27 cycles) – sense primer, 5′-ACGATGAATGTGGCGATACT-3′ and antisense primer, 5′-AGTGCATTCAAGGCTGTTGG-3′ NRP-2 (407 bp, 30 cycles) – sense primer, 5′-ATTCGATGGTCCCGTTGG-3′ and antisense primer, 5′-ACCGTGCGGAGGTCGTTT-3′ actin – sense primer, 5′-GGAGTACTTCCGCTCAGG-3′ and antisense primer, 5′-GCACTCTTCCAGCCTTCCT-3′.

### Preparation of conditioned medium

Conditioned media were prepared as follows: cells were grown in 1% FBS–MEM for 72 h, then CM was collected, centrifuged to remove cell debris and concentrated about 20-fold (20 × CM) by using Centricon Centrifugal Filters (Millipore Corporation) according to the manufacturer's instructions.

### Cell growth assay

The growth rate of control and Bev-A cells was evaluated with a 3-(4,5-dimethylthiazol-2-yl)-2,5-diphenyltetrazolium bromide (MTT, Sigma-Aldrich) assay. Cells were plated in 96-well plates at 2000 cells per well with MEM+5% FBS with or without Bev, and cells were incubated for 24, 48 or 72 h. The MTT assay was carried out according to the manufacturer's protocol. Absorption was read at 570 nm.

### Western blot hybridisation

Cells were solubilised in 20 mM Na-phosphate (pH 7.4), 150 mM NaCl, 1% Triton X-100, 1 mM Na_3_VO_4_, 5 mM EDTA and one complete mini-protease inhibitor cocktail tablet (per 10 ml of lysis buffer; Roche Diagnostics, Indianapolis, IN, USA). Cell lysates were then separated on 8% sodium dodecyl sulphate–polyacrylamide gels and transferred to polyvinylidene difluoride membranes (Amersham, Arlington Heights, IL, USA). Membranes were probed with primary antibodies overnight at 4°C, and the next day, they were washed thrice for 10 min with TBS and 0.1% Tween-20 and re-probed with the appropriate secondary antibody for 1 h at room temperature. After incubation and three washes, immunostained proteins were detected by Pierce ECL Western Blotting Substrate (Thermo Scientific, Rockford, IL, USA). Human umbilical vein endothelial cells served as a positive control. Vinculin served as a protein loading control.

### VEGFR inhibition studies

1 × 10^6^ control or Bev-A cells were plated in a 10 cm dish in MEM+5% FBS and allowed to attach overnight. The medium was then replaced with MEM+5% FBS with or without SU5416 (5 *μ*M) for 48 h. Whole-cell lysates were prepared, and total and phosphorylation of VEGFR-1 were assessed by western blot analysis. SU5416 was dissolved in DMSO.

### Migration and invasion assays

Migration assay was conducted as described previously (Dallas *et al*, 2008) with minor modifications. Equal numbers (125 000 cells per well) of control or Bev-A cells were suspended in 0.5 ml of 1% MEM–FBS with or without Bev and placed in the top compartment of a standard 8-*μ*m-pore Boyden chamber; 0.750 ml of 10% MEM–FBS with or without Bev was added to the bottom compartment. Following 48-h incubation under standard conditions (37°C/5% CO_2_), non-migrating cells were scraped from the top compartment, and cells that had migrated to the bottom compartment were fixed and stained using the Protocol HEMA 3 stain set (Thermo Fisher Scientific, Pittsburgh, PA, USA). Membranes were excised and mounted on a standard microscope slide (Curtis Matheson Scientific, Houston, TX, USA). The numbers of migrated cells were determined from five random high-power fields visualised at × 200 magnification.

Invasion assays used identical methods, except that the cells were placed in the top compartment of a modified Boyden chamber on a Matrigel-coated membrane. The numbers of invasive cells were determined from five random fields visualised at × 200 magnification.

### Scratch wound-healing/migration assay

The scratch assay was used to further evaluate the difference in migration between the control and Bev-A cell lines. Cell lines were grown in 10-cm plates, and when cells were 90% confluent, scratches were made on the plates using a sterile 200-*μ*l pipette tip. The plates were then carefully washed twice with phosphate-buffered saline. Fresh culture medium supplemented with 5% FBS with or without Bev was gently added onto the plates. The plates were allowed to incubate over the next 1–2 days. Photographs were taken at regular intervals to monitor closure of the gap. Image J 1.43u/Java 1.6.0_10 (32-bit) analysis software from the NIH is used to quantify the results of wound closure.

### *In vivo* metastasis study

To evaluate the metastatic potential of Bev-A cells, HCT116/control and HCT116/Bev-A cells were stably infected with a luciferase reporter gene lentiviral construct. A total of 1.5 × 10^6^ luciferase-labelled cells were suspended in 100 *μ*l of HBSS and injected intravenously via the tail vein. The incidence and volume of metastasis was estimated by serially imaging mice for bioluminescence using the IVIS 100 imaging system coupled to a data-acquisition personal computer equipped with the Living Image software (Xenogen, Hopkinton, MA, USA). The mice were anaesthetised with a 1.5% isoflurane–oxygen mixture and injected intraperitoneally with luciferase potassium salt solution (Sigma-Aldrich) at a dose of 150 mg kg^−1^ body weight, waiting for 10 min before imaging. The photon emission (representative of luciferase activity) was used to assess relative tumour burden in the mice.

### Statistical analyses

For the *in vitro* studies, statistical analyses were carried out using the Student's *t*-test. For the *in vivo* studies, statistical significance was determined by using the Fisher's exact test (comparison of incidence) or Mann–Whitney *U*-test (comparison of means), as indicated. All statistical tests were two-sided, and *P*-values <0.05 were considered to be statistically significant.

## Results

### Chronic Bev exposure increased VEGF family members

**Figure 1:**
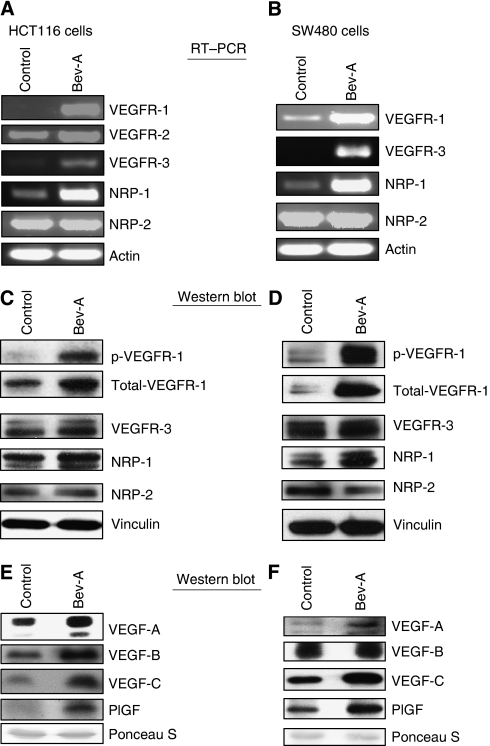
Effect of chronic bevacizumab exposure on CRC cells VEGF family profile. (**A** and **B**) RT–PCR shows that cells under Bev treatment have increased VEGFR-1, -3 and NRP-1 expression in both HCT116 and SW480 cells. (**C** and **D**) Western blot confirmed that in both HCT116 and SW480 cells, chronic bevacizumab exposure increased phosphorylation and total level of VEGFR-1 expression. Vascular endothelial growth factor receptor-3 and NRP-1, -2 remained unchanged in HCT116 cells. Neuropilin-1 was increased and VEGFR-3 and NRP-2 remained unchanged in SW480 cells. (**E** and **F**) Conditioned medium was collected and western blot performed in both HCT116 and SW480 cell. Cells under bevacizumab exposure had increased VEGF-A, -C and PlGF expression. Vascular endothelial growth factor-B was increased in HCT116 cells and remained unchanged in SW480 cells.

To study the effects of chronic Bev exposure on VEGF family members in CRC cells, the expression of VEGFRs and ligands were determined by reverse transcription (RT)–PCR and western blotting in control (IgG) and Bev-A cell lines. Both HCT116/Bev-A and SW480/Bev-A cell lines showed increased VEGFR-1, -3 and NRP-1 expression compared with the respective control cells by RT–PCR (Figures 1A and B). Vascular endothelial growth factor receptor-2 levels did not change in HCT116 cells and was undetectable in SW480 cells. Neuropilin-2 did not change in both of HCT116 and SW480 cell lines. These findings were further studied at the protein level by western blot analysis. Both HCT116/Bev-A and SW480/Bev-A cell lines showed increased expression of total and phospho-VEGFR-1. Neuropilin-1 protein level was increased in SW480/Bev-A cells only. The levels of total VEGFR-3 and NRP-2 protein expression remained unchanged in both cell lines (Figures 1C and D). There was no difference in VEGFR-2 phosphorylation and total level of protein expression in HCT116 cells (data not shown). Conditioned medium was collected from the cells and western blots were carried out to determine levels of VEGF family ligands. Both HCT116/Bev-A and SW480/Bev-A cell lines showed significantly increased VEGF-A, -C and PlGF protein levels. Ponceau S served as loading control. Vascular endothelial growth factor-B was increased in HCT116/Bev-A cells and remained unchanged in SW480 cells (Figures 1E and F).

### Chronic Bev exposure enhances CRC cell migration

**Figure 2:**
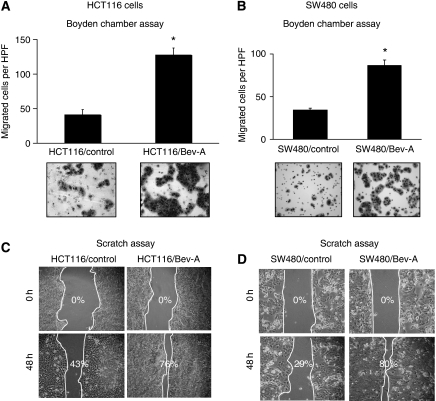
Effect of chronic bevacizumab exposure on CRC cell migration. (**A** and **B**) Using Boyden chamber migration assays, both HCT116/Bev-A (**A**) and SW480/Bev-A (**B**) cells showed a two- to three-fold increase in migration compared with that of control cells (*P*<0.001, *P*<0.0001, respectively). (**C** and **D**) Using the scratch assay, both HCT116/Bev-A (**C**) and SW480/Bev-A (**D**) cells showed an increase in migration compared with control cells (76 *vs* 43% 80 *vs* 29%, respectively). Wound closure was more complete in the Bev-A cells than in control cells.

To study the effects of chronic Bev exposure on cell migration, control and Bev-A CRC cells were seeded in a modified Boyden chamber with or without Bev, and migration was assessed 48 h later. Both HCT116/Bev-A and SW480/Bev-A cell lines showed a three- or two-fold increase in migration compared with the respective control cells (Figure 2A, *P*<0.001 *vs* control; Figure 2B, *P*<0.0001 *vs* control). Both HCT116/Bev-A and SW480/Bev-A cell lines exhibited growth rates similar to those of their respective controls, as determined by MTT assay (data not shown). To further confirm the result from the Boyden chamber assay, motility of the Bev-A cells was assessed by the scratch assay (wound healing assay). In the scratch assay, the Bev-A cells migrated inwardly and covered a greater area of the scratch at 48 h than did control cells. Similar results were observed for both HCT116/Bev-A and SW480/Bev-A cells (Figures 2C and D). (HCT116/Bev-A 76% *vs* HCT116/control 43% SW480/Bev-A 80% *vs* SW480/control 29%).

### Chronic Bev exposure enhances CRC cell invasion

**Figure 3:**
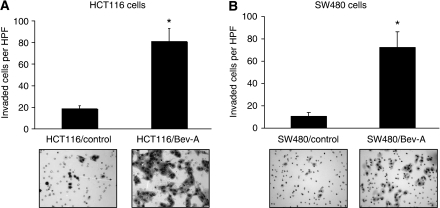
Effect of chronic bevacizumab exposure on CRC cell invasion. (**A**) Using modified Boyden chamber assays, HCT116/Bev-A showed a three- to four-fold increase in invasion compared with the HCT116/control cells (*P*<0.001). (**B**) SW480/Bev-A showed a four- to five-fold increase in invasion compared with the SW480/control cells (*P*<0.0004).

Next, the Bev-A cells were evaluated for their invasive capabilities. In Matrigel invasion assays, both HCT116/Bev-A and SW480/Bev-A cells showed increased invasion (three- to four-fold) compared with the respective control cells (Figure 3A, *P*<0.01 *vs* control; Figure 3B, *P*<0.0004 *vs* control).

### SU5416 inhibited CRC cell migration

**Figure 4:**
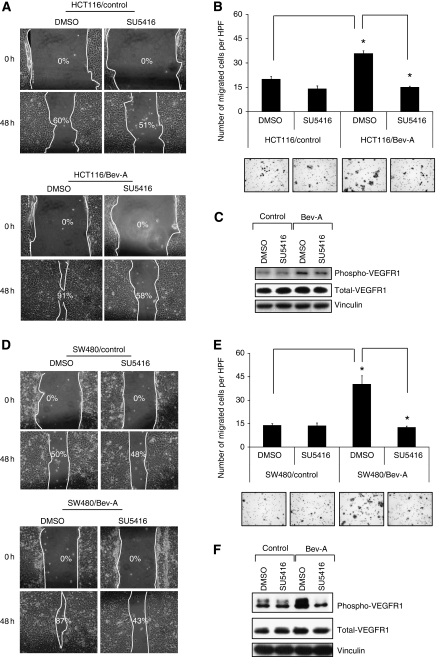
Effect of chronic bevacizumab exposure on CRC cell migration under SU5416 treatment. SU5416 treatment led to decreased cell migration in HCT116/Bev-A cells determined by *in vitro* wound healing/migration assay (**A**) and the modified Boyden chamber assay (**B**) (*P*<0.0002). (**C**) Western blots showed a decrease in VEGFR-1 phosphorylation with SU5416 treatment in HCT116/Bev-A compared with control. Vinculin served as a loading control. (**D**–**F**) These results were further confirmed in the second cell line, SW480/Bev-A cells (*P*<0.001).

To test the effect of inhibition of VEGFR-1 activation on cell migration, motility of the Bev-A cells was assessed by the scratch assay in the presence or absence of SU5416. As above, both HCT116/Bev-A and SW480/Bev-A cells migrated inwardly and covered a greater area of the defect than did control cells (HCT116/Bev-A 91% *vs* HCT116/control 60% SW480/Bev-A 87% *vs* SW480/control 50%). Treatment with SU5416 blocked cell migration (Figures 4A and D) (HCT116/Bev-A+SU5416 58% *vs* HCT116/Bev 91% SW480/Bev-A+SU5416 43% *vs* SW480/Bev-A 87%). To further confirm the result from the scratch assay. HCT116/Bev-A and SW480/Bev-A cells were pretreated with or without SU5416 (5 *μ*M) for 4 h, cells were then trypsinised and seeded in a modified Boyden chamber with or without SU5416 for 48 h. As above, both HCT116/Bev-A and SW480/Bev-A cell lines showed a two- to three-fold increase in migration compared with the respective control cells (*P*<0.0002 *vs* control; *P*<0.001 *vs* control, respectively). The Bev-A cells treated with SU5416 showed decreased migration compared with solvent alone (Figures 4B and E, *P*<0.000004 *vs* HCT116/Bev-A+DMSO; *P*<0.001 *vs* SW480/Bev-A+DMSO). Both HCT116/Bev-A and SW480/Bev-A cell lines exhibited growth rates similar to those of their respective controls, as determined by MTT assay (data not shown). Chronic exposure to Bev led to an increase in the level of phosphorylated VEGFR-1 in the both of HCT116/Bev-A and SW480/Bev-A cells. Treatment of HCT116/Bev-A and SW480/Bev-A cells with SU5416 led to decreased expression of phosphorylated VEGFR-1 as determined by western blotting (Figures 4C and F).

### Bev-A cells increased metastatic potential *in vivo*

**Figure 5:**
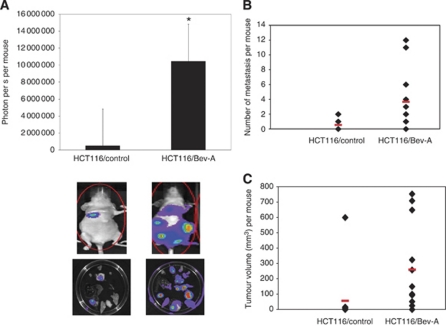
Effect of chronic bevacizumab exposure on metastatic potential of CRC cells *in vivo*. (**A**) The mice injected with HCT116/Bev-A cells showed significantly higher luciferase activity (∼10-fold higher) compared to the mice injected with control cells (*P*<0.005). Images of the whole animal and harvested organs revealed that luciferase activity was higher from organs harvested in mice injected with the HCT116/Bev-A cells (lower panel). (**B**) There was a significant increase in the mean number of metastases in the Bev-A group compared with the control group (*P*<0.02, red hash mark represents the mean). (**C**) Bev-A cells injected intravenously led to a significant increase in average tumour volume per mouse compared with control cells (*P*<0.01, red hash mark represents the mean). The colour reproduction of this figure is available on the html full text version of the manuscript

Because migration and invasion are theoretically associated with the metastatic phenotype, luciferase labelled HCT116/control and HCT116/Bev-A cells were injected into the tail vein of athymic nude mice. At the end of 6 weeks, all mice were killed. The mice injected with HCT116/Bev-A cells had a higher incidence of metastasis than that in mice injected with control cells (10 out of 12 Bev *vs* 4 out of 11 control, *P*<0.05). Mice injected with HCT116/Bev-A cells showed significantly higher luciferase activity (∼10-fold higher) compared with the mice injected with control cells (*P*<0.005, Figure 5A, upper panel). Imaging of the whole animal and harvested organs revealed that luciferase activity was higher in almost every organ harvested in mice injected with the HCT116/Bev-A cells (Figure 5A, lower panel). All mice underwent a detailed necropsy and all masses were removed to calculate the average number of metastases. There was a significant increase in the total number of metastases in the Bev-A group compared with the control group (Figure 5B, *P*<0.02). In addition, there was a significant increase in total tumour burden in the Bev-A group as assessed by adding the volume of each metastasis for each mouse in each group (Figure 5C, *P*<0.01).

## Discussion

Despite the proven clinical benefit of the VEGF monoclonal antibody Bev (with chemotherapy) in prolonging progression-free survival of patients with metastatic CRC, the benefit of therapy is short-lived (∼1–4 months), and the vast majority of patients eventually progress (Hurwitz *et al*, 2004; Giantonio *et al*, 2007; Saltz *et al*, 2008). Preclinical studies and clinical observations have now begun to shed light on the mechanisms of action of anti-VEGF therapy and acquired resistance (reviewed in Bergers and Hanahan (2008) and Ellis and Hicklin (2008, 2009)).

Owing to the role of VEGF in mediating multiple components of the tumour microenvironment, mechanisms of acquired resistance are complex and multi-factorial. For example, Ebos *et al* (2007) showed that administration of a VEGFR tyrosine kinase inhibitor to non-tumour-bearing mice led to an increase in levels of circulating cytokines such as granulocyte colony-stimulating factor, SDF-1*α*, stem cell factor and osteopontin. This result shows the role of adaptation of the host vasculature in response to blockade of VEGF signalling. Recently, several publications have reported that VEGFR blockade can lead to an increase in tumour invasion and metastasis (Ebos *et al*, 2009; Paez-Ribes *et al*, 2009). These publications raised a great deal of interest and concern in the oncology community, as four VEGF-targeted agents are now approved for use in the United States in patients with advanced-stage or metastatic malignancies. The mechanism for these observations remains to be elucidated, but it has been hypothesised that the adaptive response to blockade of VEGF signalling leads to a compensatory increase in cytokines that may increase tumour aggressiveness (Ellis and Hicklin, 2009). However, it is important to point out that these investigators utilised single-agent VEGF-targeted therapies, without the addition of chemotherapy. Chemotherapy, in and of itself, can lead to alterations in cytokine signalling (Fan *et al*, 2008), making preclinical modelling of clinical disease a challenge (Ellis and Fidler, 2010). Sorting out the effects of VEGF inhibition on the tumour microenvironment poses challenges because multiple cell types within the tumour microenvironment express VEGFRs. These cell types include endothelial cells, pericytes, immune effector cells and tumour cells themselves. One approach to understanding the contributions of individual cellular components of the tumour is to create VEGF-resistant/adapted cells *in vitro*, and study their phenotype *in vivo*. In our studies, we focused on the direct effects of Bev adaptation on CRC cells, in contrast to other studies that have focused entirely on endothelial cells.

Our studies showed that CRC cells exposed to Bev *in vitro* showed marked molecular and phenotypic changes. Bevacizumab adaptation resulted in increased migration at 1 week, 1 month, 2 months (data not shown) and 3 months (the time point used for all studies shown in this manuscript) and invasion of human CRC cell lines *in vitro*; however, proliferation remained unaffected. As expected, the increase in invasion and migration of Bev-adapted cells led to an increase in *in vivo* metastatic potential. We found no morphological evidence of EMT by molecular markers or morphological alterations. As many studies have shown that blockade of VEGF signalling *in vivo* leads to compensatory increases in the expression of VEGF family members, we investigated alterations in the VEGF family of ligands. We also investigated changes in VEGFR level and activation on tumour cells, as we have previously shown that VEGFRs are present and functional on tumour cells. Our studies showed that blockade of autocrine VEGF signalling in tumour cells led to induction of VEGF and other VEGF family members. We found a marked induction of VEGFR-1 levels and activation of VEGFR-1, whereas there were no changes in VEGFR-3 levels; VEGFR-2 levels and phosphorylation status did not change in HCT116 cells and was undetectable in SW480 cells (and therefore the increased levels of VEGF-C would not effect VEGFR-2 activation in SW480 cells). As VEGFR-1 was activated (likely due to the observed induction of PlGF and VEGF-B, two VEGF family members that bind to VEGFR-1), we sought to determine if the increase in VEGFR-1 activation mediated the increase in migration. Previously, we have shown that VEGFR-1 activation mediates migration in CRC cells (Fan *et al*, 2005). The tyrosine kinase inhibitor SU5416 primarily inhibits activation of the VEGF tyrosine kinase receptors, with some activity to other related kinases including Kit and Ret (Fong *et al*, 1999). SU5416 blocked the induction of VEGFR-1 observed in Bev-adapted cells and likewise inhibited the increase in migration observed in these cells. Cumulatively, the above studies show that CRC cells exhibit autocrine VEGF/VEGFR signalling and inhibition of VEGF signalling leads to compensatory pathways mediated via VEGFR-1 activation that lead to increased migration and invasion.

This observed increase in tumour cell invasion, migration and metastasis with VEGF signalling inhibition is intriguing considering the recent work of others suggesting that VEGF inhibition in mice can increase metastasis (Casanovas *et al*, 2005; Ebos *et al*, 2009). However, it is important to consider these studies in light of results from several clinical trials in CRC. In the clinic, patients with metastatic CRC are treated with Bev in combination with chemotherapy, but Bev is not approved for use as a single agent in the United States. No study has show an increase in rates of metastasis in patients with metastatic CRC treated with Bev and chemotherapy (Ellis and Reardon, 2010). However, a few clinical studies have examined the use of Bev as a single agent in metastatic CRC, and the one study conducted with single-agent Bev in such patients did not specifically investigate sites of metastasis or reasons for disease progression (Giantonio *et al*, 2007). However, there are now two completed Phase III trials studying the use of Bev and chemotherapy in the adjuvant setting in patients with stage 2 and 3 cancer. Both trials had a similar trial design: chemotherapy alone *vs* chemotherapy and Bev for ∼6 months, followed by single-agent Bev for 6 months. The first trial, National Surgical Adjuvant Breast and Bowel Project CO-8, was recently published (Allegra *et al*, 2011). In this study, there was no benefit to patients who received Bev in addition to chemotherapy, as disease-free survival was the same for both groups at 3 years. In the AVANT trial, there was likewise no benefit when Bev was added to chemotherapy, and interestingly, patients who received chemotherapy alone appeared to have a better outcome than those patients who received chemotherapy and Bev (de Gramont *et al*, 2011). In both studies, the per cent of patients who recurred at multiple sites of metastasis was similar. However, in light of the AVANT trial outcome where the patients who received Bev seemed to have a shorter disease-free survival, one must scrutinise the results of other clinical trials in the adjuvant setting where single-agent VEGF-targeted therapies are utilised. It is clear that the effect of VEGF-targeted therapy in the adjuvant setting is not the same as when used in patients with documented metastatic CRC.

Our studies showing that *in vitro* Bev adaptation led to an increase in tumour aggressiveness *in vitro* show two important findings. The first is that VEGFRs are present and functional on CRC cells, as blockade leads to a phenotypic change. The second is that blockade of VEGF signalling as a single agent in CRC cells can possibly lead to an increase in tumour cell aggressiveness. Therefore, the use of single-agent Bev in patients with CRC should not be considered outside of a clinical trial setting.

In conclusion, chronic blockade of VEGF signalling in CRC by Bev leads to an increase in cell migration and invasion *in vitro*, and metastasis *in vivo.* These findings may explain, in part, the studies by others showing that VEGF blockade *in vivo* leads to an increase in metastatic potential. On the basis of these studies and clinical trial results, there is no justification at this time to use single-agent Bev in patients with metastatic CRC. However, these studies were carried out with single-agent Bev and do not take into account the complexities of combining Bev with chemotherapy *in vivo*. Interestingly, the *in vitro* increase in tumour cell migration and invasion in Bev-A cells was abrogated by VEGFR1 inhibition, suggesting that perhaps this combination should be further explored in preclinical studies and, if appropriate, in clinical studies.

## References

[bib1] Allegra CJ, Yothers G, O’Connell MJ, Sharif S, Petrelli NJ, Colangelo LH, Atkins JN, Seay TE, Fehrenbacher L, Goldberg RM, O’Reilly S, Chu L, Azar CA, Lopa S, Wolmark N (2011) Phase III trial assessing bevacizumab in stages II and III carcinoma of the colon: results of NSABP protocol C-08. J Clin Oncol 29: 11–16 20940184 10.1200/JCO.2010.30.0855 PMC3055856

[bib2] Batchelor TT, Sorensen AG, di Tomaso E, Zhang WT, Duda DG, Cohen KS, Kozak KR, Cahill DP, Chen PJ, Zhu M, Ancukiewicz M, Mrugala MM, Plotkin S, Drappatz J, Louis DN, Ivy P, Scadden DT, Benner T, Loeffler JS, Wen PY, Jain RK (2007) AZD2171, a pan-VEGF receptor tyrosine kinase inhibitor, normalizes tumor vasculature and alleviates edema in glioblastoma patients. Cancer Cell 11: 83–95 17222792 10.1016/j.ccr.2006.11.021 PMC2748664

[bib3] Bergers G, Hanahan D (2008) Modes of resistance to anti-angiogenic therapy. Nat Rev Cancer 8: 592–603 18650835 10.1038/nrc2442 PMC2874834

[bib4] Casanovas O, Hicklin DJ, Bergers G, Hanahan D (2005) Drug resistance by evasion of antiangiogenic targeting of VEGF signaling in late-stage pancreatic islet tumors. Cancer Cell 8: 299–309 16226705 10.1016/j.ccr.2005.09.005

[bib5] Dallas NA, Gray MJ, Xia L, Fan F, van Buren II G, Gaur P, Samuel S, Lim SJ, Arumugam T, Ramachandran V, Wang H, Ellis LM (2008) Neuropilin-2-mediated tumor growth and angiogenesis in pancreatic adenocarcinoma. Clin Cancer Res 14: 8052–8060 19088020 10.1158/1078-0432.CCR-08-1520

[bib6] de Gramont A, Van Cutsem E, Tabernero J, Moore MJ, Cunningham D, Rivera F, Im SA, Makrutzki M, Shang A, Hoff PM (2011) AVANT: results from a randomized, three-arm multinational phase III study to investigate bevacizumab with either XELOX or FOLFOX4 *vs* FOLFOX4 alone as adjuvant treatment for colon cancer. Proceedings of 2011 Gastrointestinal Cancers Symposium, San Francisco, CA

[bib7] Ebos JM, Lee CR, Christensen JG, Mutsaers AJ, Kerbel RS (2007) Multiple circulating proangiogenic factors induced by sunitinib malate are tumor-independent and correlate with antitumor efficacy. Proc Natl Acad Sci USA 104: 17069–17074 17942672 10.1073/pnas.0708148104 PMC2040401

[bib8] Ebos JM, Lee CR, Cruz-Munoz W, Bjarnason GA, Christensen JG, Kerbel RS (2009) Accelerated metastasis after short-term treatment with a potent inhibitor of tumor angiogenesis. Cancer Cell 15: 232–239 19249681 10.1016/j.ccr.2009.01.021 PMC4540346

[bib9] Ellis LM, Fidler IJ (2010) Finding the tumor copycat: therapy fails, patients don’t. Nat Med 16: 974–975 20823880 10.1038/nm0910-974

[bib10] Ellis LM, Hicklin DJ (2008) VEGF-targeted therapy: mechanisms of anti-tumour activity. Nat Rev Cancer 8: 579–591 18596824 10.1038/nrc2403

[bib11] Ellis LM, Hicklin DJ (2009) Resistance to targeted therapies: refining anticancer therapy in the era of molecular oncology. Clin Cancer Res 15: 7471–7478 20008847 10.1158/1078-0432.CCR-09-1070

[bib12] Ellis LM, Reardon DA (2010) Is there really a yin and yang to VEGF-targeted therapies? Lancet Oncol 11: 809–811 20634133 10.1016/S1470-2045(10)70161-3

[bib13] Fan F, Gray MJ, Dallas NA, Yang AD, Van Buren II G, Camp ER, Ellis LM (2008) Effect of chemotherapeutic stress on induction of vascular endothelial growth factor family members and receptors in human colorectal cancer cells. Mol Cancer Ther 7: 3064–3070 18790786 10.1158/1535-7163.MCT-08-0615 PMC2581833

[bib14] Fan F, Wey JS, McCarty MF, Belcheva A, Liu W, Bauer TW, Somcio RJ, Wu Y, Hooper A, Hicklin DJ, Ellis LM (2005) Expression and function of vascular endothelial growth factor receptor-1 on human colorectal cancer cells. Oncogene 24: 2647–2653 15735759 10.1038/sj.onc.1208246

[bib15] Ferrara N (2009) Vascular endothelial growth factor. Arterioscler Thromb Vasc Biol 29: 789–791 19164810 10.1161/ATVBAHA.108.179663

[bib16] Folkins C, Shaked Y, Man S, Tang T, Lee CR, Zhu Z, Hoffman RM, Kerbel RS (2009) Glioma tumor stem-like cells promote tumor angiogenesis and vasculogenesis via vascular endothelial growth factor and stromal-derived factor 1. Cancer Res 69: 7243–7251 19738068 10.1158/0008-5472.CAN-09-0167 PMC3409689

[bib17] Fong TA, Shawver LK, Sun L, Tang C, App H, Powell TJ, Kim YH, Schreck R, Wang X, Risau W, Ullrich A, Hirth KP, McMahon G (1999) SU5416 is a potent and selective inhibitor of the vascular endothelial growth factor receptor (Flk-1/KDR) that inhibits tyrosine kinase catalysis, tumor vascularization, and growth of multiple tumor types. Cancer Res 59: 99–106 9892193

[bib18] Giantonio BJ, Catalano PJ, Meropol NJ, O’Dwyer PJ, Mitchell EP, Alberts SR, Schwartz MA, Benson III AB (2007) Bevacizumab in combination with oxaliplatin, fluorouracil, and leucovorin (FOLFOX4) for previously treated metastatic colorectal cancer: results from the Eastern Cooperative Oncology Group Study E3200. J Clin Oncol 25: 1539–1544 17442997 10.1200/JCO.2006.09.6305

[bib19] Herbst RS, Johnson DH, Mininberg E, Carbone DP, Henderson T, Kim ES, Blumenschein Jr G, Lee JJ, Liu DD, Truong MT, Hong WK, Tran H, Tsao A, Xie D, Ramies DA, Mass R, Seshagiri S, Eberhard DA, Kelley SK, Sandler A (2005) Phase I/II trial evaluating the anti-vascular endothelial growth factor monoclonal antibody bevacizumab in combination with the HER-1/epidermal growth factor receptor tyrosine kinase inhibitor erlotinib for patients with recurrent non-small-cell lung cancer. J Clin Oncol 23: 2544–2555 15753462 10.1200/JCO.2005.02.477

[bib20] Hicklin DJ, Ellis LM (2005) Role of the vascular endothelial growth factor pathway in tumor growth and angiogenesis. J Clin Oncol 23: 1011–1027 15585754 10.1200/JCO.2005.06.081

[bib21] Hurwitz H, Fehrenbacher L, Novotny W, Cartwright T, Hainsworth J, Heim W, Berlin J, Baron A, Griffing S, Holmgren E, Ferrara N, Fyfe G, Rogers B, Ross R, Kabbinavar F (2004) Bevacizumab plus irinotecan, fluorouracil, and leucovorin for metastatic colorectal cancer. N Engl J Med 350: 2335–2342 15175435 10.1056/NEJMoa032691

[bib22] Kaplan RN, Riba RD, Zacharoulis S, Bramley AH, Vincent L, Costa C, MacDonald DD, Jin DK, Shido K, Kerns SA, Zhu Z, Hicklin D, Wu Y, Port JL, Altorki N, Port ER, Ruggero D, Shmelkov SV, Jensen KK, Rafii S, Lyden D (2005) VEGFR1-positive haematopoietic bone marrow progenitors initiate the pre-metastatic niche. Nature 438: 820–827 16341007 10.1038/nature04186 PMC2945882

[bib23] Paez-Ribes M, Allen E, Hudock J, Takeda T, Okuyama H, Vinals F, Inoue M, Bergers G, Hanahan D, Casanovas O (2009) Antiangiogenic therapy elicits malignant progression of tumors to increased local invasion and distant metastasis. Cancer Cell 15: 220–231 19249680 10.1016/j.ccr.2009.01.027 PMC2874829

[bib24] Poesen K, Lambrechts D, Van Damme P, Dhondt J, Bender F, Frank N, Bogaert E, Claes B, Heylen L, Verheyen A, Raes K, Tjwa M, Eriksson U, Shibuya M, Nuydens R, Van Den Bosch L, Meert T, D’Hooge R, Sendtner M, Robberecht W, Carmeliet P (2008) Novel role for vascular endothelial growth factor (VEGF) receptor-1 and its ligand VEGF-B in motor neuron degeneration. J Neurosci 28: 10451–10459 18923022 10.1523/JNEUROSCI.1092-08.2008 PMC6671326

[bib25] Saltz LB, Clarke S, Diaz-Rubio E, Scheithauer W, Figer A, Wong R, Koski S, Lichinitser M, Yang TS, Rivera F, Couture F, Sirzen F, Cassidy J (2008) Bevacizumab in combination with oxaliplatin-based chemotherapy as first-line therapy in metastatic colorectal cancer: a randomized phase III study. J Clin Oncol 26: 2013–2019 18421054 10.1200/JCO.2007.14.9930

[bib26] Shin JY, Yoon IH, Kim JS, Kim B, Park CG (2009) Vascular endothelial growth factor-induced chemotaxis and IL-10 from T cells. Cell Immunol 256: 72–78 19249018 10.1016/j.cellimm.2009.01.006

[bib27] Wey JS, Fan F, Gray MJ, Bauer TW, McCarty MF, Somcio R, Liu W, Evans DB, Wu Y, Hicklin DJ, Ellis LM (2005) Vascular endothelial growth factor receptor-1 promotes migration and invasion in pancreatic carcinoma cell lines. Cancer 104: 427–438 15952180 10.1002/cncr.21145

